# Family experiences of inpatient mental health care for people with dementia

**DOI:** 10.3389/fpsyt.2023.1093894

**Published:** 2023-03-01

**Authors:** Emma L. Wolverson, Karen Harrison Dening, Rebecca Dunning, George Crowther, Gregor Russell, Benjamin R. Underwood

**Affiliations:** ^1^Faculty of Health Sciences, University of Hull, Hull, United Kingdom; ^2^Dementia UK, London, United Kingdom; ^3^Humber Teaching NHS Foundation Trust, Hull, United Kingdom; ^4^Leeds and York Partnership NHS Foundation Trust, Leeds, United Kingdom; ^5^Leeds Institute of Health Science, University of Leeds, Leeds, United Kingdom; ^6^Bradford District Care NHS Foundation Trust, Osprey House, Lynfield Mount Hospital, Bradford, United Kingdom; ^7^Cambridgeshire and Peterborough NHS Foundation Trust, Windsor Unit, Fulbourn Hospital, Cambridge, United Kingdom; ^8^Department of Psychiatry, University of Cambridge, Cambridge, United Kingdom

**Keywords:** dementia, mental health, inpatient, experience, family carer

## Abstract

**Introduction:**

This study investigates family carers experiences of inpatient mental health care for people with dementia. A mental health inpatient admission for a person with dementia is usually considered when a person is distressed and this distress leads to behaviours that are assessed to be risky for the person or others.

**Methods:**

Participants included seven family carers whose relative with dementia had been cared for within a mental health ward in the United Kingdom UK within the last 5 years. Interviews were used to explore the circumstances that led to the admission, expectations of mental health care, and perceptions of care during the admission and support received by family carers.

**Results:**

Participants described their distress at the time of admission and how the process of admission sometimes added to their distress. Carers often felt excluded from their relatives care in hospital and many felt a loss of their rights. Participants felt that the mental health admission had a negative impact on their relative with dementia. Mental health law and legislation was difficult to navigate and carers were concerned about how much knowledge and understanding of dementia staff have.

**Discussion:**

Findings suggest that family carers may benefit from targeted support during their relatives admission. Mental health wards could do more to recognise the expertise in care and knowledge of the person with dementia of family carers and involve them in planning care. More research is needed to explore the experiences and outcomes of people with dementia during such admissions.

## 1. Introduction

Distress is one of the most common clinical manifestations of dementia and a significant issue for people with dementia and their family carers (1). Distressed behavior can take many forms, including agitation, apathy and sleep disturbance. Historically terms like “challenging behavior” or “behavior that challenges” have been used to describe behavior changes associated with dementia, but these terms are disliked by people with dementia (2), therefore in this paper we use the term distress to refer to behavioral changes associated with dementia. Irrespective of how distress manifests, it is estimated to occur in nearly 80% of people living with dementia (3). When a person with dementia is distressed, their quality of life deteriorates, they have more unplanned hospital admissions and they are more likely to experience an earlier move into residential care (4). Distressed behavior also has significant effects on caregiver wellbeing, increasing exhaustion and depression (5).

Historically distress in dementia has often been managed through the use of sedative medications, although policy and best practice guidelines advocate non-pharmacological approaches as first-line treatments (6). Some people with dementia may show their distress through actions which are considered risky or harmful to themselves or others, on these occasions a person may be admitted to a mental health or psychiatric hospital with the aim of providing intensive levels of assessment, monitoring and treatment that is not possible in other settings (7). In the United Kingdom, the majority of people with dementia admitted to mental health wards are subject to compulsory treatment under the Mental Health Act (1983) as they are unable to consent to their admission and through their actions are taken to be objecting to care. A person can be detained for assessment if health professionals think they are behaving in a way that puts their health at risk, or if they are a danger to themselves or others (Mental Health Act, 1983). People may be admitted to a specialist ward just for people with dementia or in an older peoples ward alongside people with other mental health difficulties (7).

International research shows that people with dementia within mental health wards are a very vulnerable population as they are often in the advanced stage of dementia with high levels of co-morbidity and frailty (8). There is very little published research on the quality of care or outcomes for people with dementia following an admission to a mental health ward, and to date there have been no qualitative studies exploring the perspectives or experiences of people with dementia or their families.

Concerns have been raised about the psychological trauma experienced by people with dementia and their families during an admission to a mental health ward (9). Exploring the perspectives of people with dementia during an admission may be difficult and present ethical challenges given the often advanced stage of their condition and high levels of distress experienced at this time. However, proxy experiences of such an admission through the insights of family carers could provide useful insight. Indeed, carer distress (10–13) and illness (12, 14) are common reasons cited to contribute to such an admission. While the literature suggests that family carers may benefit from education and psychological support as part of inpatient treatment (4, 15–17), it is not currently known what support is offered or, crucially, what carers needs for support are during this time.

This qualitative study enrolled family carers to address the question: what are family carers experiences of inpatient mental health admission for people with dementia’?

## 2. Methods

A qualitative study was undertaken from May to August 2022 using semi-structured individual interviews and reflexive thematic analysis. The study was approved by University of Hull Faculty of Health Sciences Ethics Committee.

### 2.1. Participants and sampling

Purposive sampling was used to recruit family carer participants according to the inclusion and exclusion criteria. As this was intended to be an exploratory study the aim was to recruit between 6 and 10 participants (18).

#### 2.1.1. Inclusion criteria

People who have experience of supporting a family member with dementia who has had an admission to an inpatient mental health unit in the United Kingdom within the last 5 years. A 5-year time frame was selected for pragmatic reasons, (1) to provide reasonable sample pool given that it is a relatively small proportion of people with dementia who are admitted to mental health wards and (2) as we were conscious that families’ experiences were likely to have been different during the COVID pandemic so wanted to expand our inclusion period before COVID. No exclusion criteria were set regarding the relationship to the person with dementia.

#### 2.1.2. Exclusion criteria

People whose relative’s received care in an inpatient setting over 5 years ago were excluded as care practices on wards will have changed over time, in the United Kingdom in 2018, the National Institute for Health and Care Excellence (NICE) published national guidelines for dementia which had recommendations for staff training and carer support which directed at all providers of health and social care for people with dementia (6). People who did not have sufficient fluency in English to take part in an interview as no budget was available for a translator.

### 2.2. Setting

The intention was to explore inpatient mental health care for people with dementia, however there is variation in provision across the United Kingdom, for example some people are admitted to specialist dementia wards and others to old age wards. People with young onset dementia are sometimes admitted to adult mental health wards as a result of their age on admission. As this was an exploratory study, no limits were set on type of mental health ward.

### 2.3. Recruitment and consent

A poster advertising the study was shared *via* social media and the Three Nations Dementia Working Group, a membership organization of individuals living with dementia across England, Northern Ireland and Wales. In addition, members of a patient and participant involvement group for people with dementia who have experienced mental health care were also approached. An information sheet was provided to everyone who expressed an interest in the research. When initial verbal consent was given, a time and date for the interview was arranged. At the interview, the participant was offered an opportunity to ask questions and written consent was taken.

### 2.4. Interviews

Semi-structured interviews were conducted to explore carers experiences of mental health inpatient care. All interviews took place online, with the exception of one which was conducted over the telephone. All interviews were audio recorded and transcribed verbatim. A topic guide for the interviews was created and is shown in Appendix A, but the interviewer adapted the phrasing and order of the questions based on participant responses, following up interesting topics raised by participants. Two of the researchers (EW and KHD) conducted the interviews; clinical psychologist and a nurse both of whom have experience of working with people with dementia and within mental health settings. Interviews lasted between 45 min and 1 h.

### 2.5. Analysis

Thematic analysis was undertaken (19). Open coding was used to ensure that descriptive codes and themes were fully supported by the data. EW and KHD independently read and re-read the transcripts to gain familiarity with the data. Both researchers worked independently initially, separating the data into meaningful segments and applying descriptive codes. Each researcher created a hierarchy of codes and interpretive themes, both engaged in constant comparison during this process to ensure the validation of the results. Then an analysis meeting was held to discuss the emerging themes, where any disagreements occurred, they were resolved by discussion, ensuring that all themes were supported by the data.

## 3. Results

Seven carers participated in the study; five females, and two males. Five were aged between 45 and 55 years and two aged between 55 and 65 years. The relationship of the carers to the person with dementia was recorded: four wives, one husband and one son and one daughter-in-law participants four and five were interviewed together. Four of the people with dementia were under the age of 65 years at the time of admission and two were over 65 years. The diagnoses of the person with dementia included Alzheimer’s disease, frontal lobe dementia, posterior cortical atrophy and cortical basal syndrome. All admissions were compulsory admissions under the Mental Health Act (1983), reasons for admission included physical aggression and agitation, paranoid thoughts, refusing care and depression. Admissions took place between 2018 and 2022. Four carers had relatives admitted during the COVID pandemic, three of these carers experienced periods of visiting restrictions and one did not.

Three themes were identified as being important to carers’ experiences: (1) the impact of the admission on carers themselves; (2) the impact of the admission on their relative with dementia; and (3) the confusion surrounding the admission. Each theme, their corresponding sub-themes, and supporting quotes are outlined below. Sub-theme titles are direct participant quotes.

### 3.1. Theme 1: Impact on family carers

The first theme describes the impact of the admission on the carer and is comprised of three subthemes: “They kind of took him away” which describes the traumatic events leading up to an admission and how the admission added to this distress; “I’ve got no rights” which captures carers feelings of exclusion; and “They just did not think about the impact on me” which relates to carers need for support.

“*They kind of took him away*”

The events leading up to an admission were incredibly traumatic for the person with dementia and for their family. Family members were often sleep deprived and exhausted, some had been attacked, threatened and feared for their safety;

There is nothing more horrific than the first time your husband turns round and hits you. He would never ever, in a million years have hit me, [before he had dementia] ever. (P01, 665–666)

… she was desperately unwell and hallucinating and things, there were people under the bed, the food they were feeding her was from poisonous sources. (P04, 113–114)

For the five of carers the process of their relative being sectioned under the Mental Health Act added to their distress and was experienced as traumatic. Because the person with dementia was often unable to fully comprehend what was happening, the process of the section sometimes involved ambulance staff and police officers and as such, the moment of the person being “removed” was a haunting memory for some carers, which was evident in the clarity of detail they shared in recalling the event;

I said don’t touch his shoes because it sets him off. She picked up his shoes, carried them and he pushed her. Then she got six more policemen to come. They restrained him. They handcuffed him. They put him on the floor … he was screaming … (P03, 243–255)

The [Mental Health Act] assessment was quite spectacular and [name of wife] did throw a table at someone, and the person that seemed most competent and most able to deal with the situation was a young woman of about 25 who I think was the note taker. (P06, 76–78)

Conversely, the distressing process of the act of detention was made better by the kindness shown by some health care professionals involved in the admissions process;

They were lovely, really good. A very positive experience for something that is actually quite traumatic. (P01, 140–141)

For some carers the process felt rushed and chaotic; for others the admission was drawn out by other events, such as a lack of beds, so that while professionals acknowledged a person required an urgent admission, this was not possible:

I had absolutely no idea. They told me the ambulance would be there in an hour and a half, […] hour to hour and a half. They gave me some plastic bags to pack [name of husband]’s clothes in […] to pack everything that he would need because I wouldn’t be allowed to see him for the first 7 days he was in the unit. (P07, 133-136)

They didn’t have a bed and therefore they couldn’t section her, they let her come home with me. We spent the whole day Friday wondering what the hell is going to happen. And they got in touch Friday evening and said ok, got a bed for her Saturday, […] and that’s the last time she’s been in this house. (P06, 89-95)

Carers spoke about their overwhelming and enduring feelings of guilt and loss at the point their relative went into hospital as an urgent admission;

There was a look of sheer terror on his face. He had no idea what was going on. (P07, 170–171)

It was that night I just absolutely went to pieces, couldn’t stop shaking and sobbing. Just could not stop. I couldn’t get over this feeling that I’d let [name of husband] down. (P07 604–606)

… thinking back [to the sectioning], was when I started all the grieving … he was picked up from here [home] and taken to a … another environment of which people wouldn’t have any idea about. (P01, 200–210)

For some, this was mixed with a sense of “relief” in that the admission might represent a turning point and that they might finally get some help. However, there was a lack of clarity about what the purpose of the admission was for or how long it might last:

I remember looking at the website [for the mental health ward] and there’s nothing, absolutely no information other than you know, a phone number and a map maybe. But we had no idea what was going to happen. (P05, 356–358)

We thought it was a care home, an NHS care home. We didn’t realize it was only there for a fixed period of time. (P04, 368–369)

“*I’ve got no rights*”

Prior to the admission carers were often providing round the clock care to support the person with dementia, whether they were living at home or in a care home. However, once the person was admitted to the mental health ward, carers often felt excluded from the person’s care. Five of carers felt that the ward did not communicate with them enough;

Then obviously [husband] disappeared off in the ambulance and that’s where the level of communication of any kind stopped. (P01, 140–143)

I was expecting them to ring me up the next morning, […] and, […] to perhaps have a form and ask in great detail about [name of husband] and his likes and dislikes and how I helped him in the home. You know, where he was at and what he could and couldn’t do and things. But I just, […] just didn’t get that. I just don’t think […] they asked for enough information (P02, 139–143)

One carer described being assertive in order to be kept updated, however, this eventually involved raising a complaint and felt this got them labeled as being a difficult relative;

It just all seemed a battle, and then I think the team got defensive and, […] and yeah. I think the psychiatrist just loathed me, really. I think she lost, […] lost it with me. (P02, 369–371)

Two carers reported good communication with their ward and as a result, felt that their relative’s condition improved quickly;

There was a pretty good communication flow between us and the ward. We had regular meetings, we went to see them in person, you know, concerns were raised, feedback was given, we had to provide our insight into mums’ background and that, like could inform how they helped to do things differently for her. So yeah, I think it settled quite quickly. (P04, 427–431)

Some carers were not able to visit the person with dementia for the entire duration of their admission, because the ward was a long distance from their home (some wards location required a commute of 3 h or more) or because of COVID restrictions, this meant some never saw the ward environment which added to their sense of fear and concerns. “Visiting” rules varied significantly between wards both during and outside of COVID restrictions. This separation caused significant distress for carers and added to their grief. Many carers spoke about their growing realization that the longer they were separated, the less likely the person with dementia was to ever return home;

I’ve got no rights. I couldn’t see him and I couldn’t get in to see him. (P02, 379–380)

There was no visiting allowed for the entire duration of his admission. For that period that he was sectioned, I didn’t see him at all … the only thing I could do, was telephone him to face time him but, as I am sure you will know, trying to face time or phone a person with quite significant Alzheimer’s is virtually impossible. So, he didn’t understand how to use a phone. (P01, 151–166)

In contrast two carers were able to visit freely, even during the COVID pandemic;

I was able to walk up and down with her and stay as long as I liked. (P06, 429–430)

Some of the participants had previously worked within the NHS and felt having an “insider” knowledge of the NHS system meant they had more understanding of the situation, these carers wondered how others, without such insights, would have managed;

We knew what to ask for, to push for and … if I hadn’t arranged a call during those first two weeks … and again, there is two of us, you know, in our fifties … but the idea of me being an 85 year old as someone in similar circumstances, trying to negotiate all of this and you know assert myself when my voice is croaky or whatever, it’s really … the machine goes on whether you go with it or not and it’s very … so, I am not saying that it’s not only the elderly but anyone can have trouble and feel disempowered by it. It’s easy for the machine to run itself but it’s about the people who are out here. (P05, 984–990)

“*They are the professionals. They know better than you*”

Some carers spoke about how their knowledge of the person and expertise in their relatives care was not considered or valued. They firmly believed that their exclusion was to the detriment of the well-being of the person with dementia;

… there’s a lot of arrogance I think in hospital care, you know? They are the professionals. They know better than you. But actually they do not know that person better than you and […] that’s the frustrating […] really with it, you know? (P03, 164–167)

One carer described their distress at listening to “experts” and appointed advocates speak about their relative in team meetings and mental health tribunals;

… this chap came up to see us and he goes, I’m [name], I’m his advocate … He goes, I know everything about him. I’ll be talking in the meeting and I’ll be telling the doctors what you need. So I said basically you’ve seen him for five minutes and you think you know his life history? And this is what we got. The advocate then proceeded to sit next to him, in the doctor’s […] in the consultant […] in the room. I couldn’t sit next to him. (P03, 121–126)

Two carers were not included in planning for their relative’s discharge from the ward;

I would ring every day, and the first that I knew was that transport was being booked in the afternoon, he was getting moved tomorrow. I had to actually ask where he was being moved to. The place he was moved to was a three hour round trip from home. (P01, 388–391)

… the [psychiatrist] said to me, if the [tribunal] panel agrees to discharge him, you’ve got to come in two hours and pick him up … I wasn’t, […] prepared. I didn’t know that beforehand and I wasn’t prepared, and that couldn’t have done it. And that’s why I wasn’t allowed to bring him home, because I wouldn’t have had a care package or any support. (P02, 421–425)

In contrast, where carers expertise was valued, carers felt involved in decision-making and respected. One carer spoke about her husband’s consultant being open to looking up research into rarer forms of dementia after she had found evidence that the medication he had been prescribed may not be suitable;

So I said, can you look at young onset literature in particular? Because I think there could be a difference in the young onset. She promised me that she would and I got an e-mail 10 o’clock that night saying [name of husband] is off Memantine tomorrow. (P7, 357–353)

“*They just did not think about the impact on me*”

All family members spoke about a lack of support for them and felt that there was a lack of any understanding of their distress or what the experience was like for them:

They just didn’t think about the impact on me. You know, I said, […] my lovely husband who could walk and talk and eat and use the toilet still, […] came out totally different and I, […] and I lost him there. I lost him. (P02, 747–750)

He’s my husband, you know, they keep forgetting the fact that he’s my husband. He’s our children’s dad. He’s our family. (P03, 559–561)

Going into the ward was also a frightening experience for families;

The only thing is, sometimes when you’ve got one of them that you know is violent, kicking off and trying to get into the room and I’m standing with my shoulder against the door to stop them getting in, it would be nice if there was some sort of button I could press. He [matron] said, there’s mobile alarms, have they never given you a mobile alarm? And I said no. And he marched straight back into the room with instructions that they had to give their visitors alarms. (P7, 442–446)

Only one carer had been in receipt of support in their own right prior to the admission, however, even this stopped once their relative became an inpatient meaning that they were then no longer eligible for the service. Several carers reported seeking private support for their own mental health needs following the admission. Many carers went on to speak about the ongoing stresses they faced after discharge from the ward;

This is our journey and it’s been quite frustrating and stressful really … and it doesn’t end. (P03, 566–567)

### 3.2. Theme 2: Impact on person with dementia from the family carers perspective

This theme reflects the carers perceptions of what the admission was like for the person with dementia, the impact of the admission felt by them and the outcomes for that person following the admission.

“*Even the nurse at the hospital said I’ve never seen somebody so distressed*”

Just as the admission to a strange environment was painful for carers, they also worried about the distress experienced by the person with dementia as a result of the separation. Carers spoke about the admission initially adding to the very distress that it was designed to reduce;

… not have access to me so he, […] he just. So I know like you said, with the assessment and things, seeing where they’re at and what they can and can’t do because he was so stressed, he was just really, […] just, […] just wouldn’t go to bed. Just screaming for me all the time. And getting more and more angry. (P02, 128–132)

Some of the carers felt that an admission initially increased a person’s distress, increased distress seemed to result in restrictive interventions, which then further added to a person’s distress;

He got more violent because he was getting restrained more (P03, 453–454).

All carers spoke about the use of antipsychotic medications to manage distress. Medication side effects like constipation and diarrhoea were viewed to add to distress;

So sometimes medication would actually cause other problems like diarrhoea which would cause more distress, which would cause more problems. (P03, 49–50)

Over-sedation was also a concern raise by some carers;

They used a lot of Diazepam and Lorazepam and, […] he just really declined very rapidly. (P02, 276–283)

So he [psychiatrist] put the risperidone up, which effectively, kind of did calm him down, but, over-calmed him as well […] so yeah like dribbling in the chair that kind of thing. (P03, 486–493)

In contrast, one carer felt the medication regime was good; “*to the best of my knowledge they were really light on sedation*”. (P04, 508–509).

Carers commented on the importance of therapeutic activities on the ward, but some expressed frustration that interventions that had managed distress at home were not implemented;

I kept saying to them, […] saying to them that he needs a long walk. He needs to get outside, which I did at home. We’d walk for an hour around the park at least, and if I didn’t do that then he wouldn’t sleep as well. I kept telling them that, but they didn’t even take him out into the garden. And then once his legs started swelling, his shoes wouldn’t fit. (P02, 571–576)

“*He lost his speech. He lost his mobility. He lost his ability to eat with a knife and fork*”

From the carers perspective outcomes of the admission for the person with dementia were very poor, carers described a rapid decline in the person’s mental and physical wellbeing. None of the people with dementia were able to return home, it remained difficult to find suitable placements for discharge and three people were readmitted to a mental health ward after discharge;

… he lost his speech. He lost his mobility. He lost his ability to eat with a knife and fork and sit at a table to eat … just lost a lot of this faculties. Yeah, […] because at home it could walk up and down. There was no issues about his mobility, he could walk up and down stairs fine. He could sit at the table or eat his dinner on a tray watching TV. No issues with that at all. And, […] and he could still talk to me, he didn’t always understand and got confused, but he could still, [use] language. (P02, 264-–72)

And we thought ok then, this will be for a period of time to get him stabilized, bring him back home, because we didn’t think care would meet his needs, and three years later we find it still doesn’t meet his needs. (P03, 69–72)

A decline in the physical health of people with dementia following an admission was mentioned by several carers. Falls and transfers from the mental health ward to the Accident and Emergency department were also seen to add to the person’s distress. Two carers attributed a decline in their relatives health to poor care practices, such as not brushing someone’s teeth and not ensuring a person was wearing their glasses;

… he was 10 stone when he first got sectioned in the first hospital. He went down to 8 stone […] 8 stone 3. (P03, 492–493)

Two bad falls, yeah. I mean, they said to me, we don’t put his glasses on because he might fall on them, […] and hurt his face. And I said, well, he’s going to fall because he can’t see. (P02, 668–670)

In contrast, one carer felt her husband’s mental state had improved;

He is much quieter and more settled. (P7, 389)

### 3.3. Theme 3: Confusion related to the admission

The mental health care system baffled many of carers who struggled to understand the law, the role and purpose of the wards.

“*I do not know the procedure*”

None of the participants had any sense of how long a person might stay in the ward;

So nobody prepared me for the road ahead. I did not anticipate, when I took her to the hospital that day, that she would never step foot into this house again. (P06, 417–419)

… it was at least a couple of weeks before we got to the idea that this was going to be for at least a couple of months, two or three months. (P05, 380–382)

Most carers had no experience of mental health services and found mental health law, the language used by professionals and the procedures baffling;

I don’t know the procedure [of the tribunal]. I don’t know the procedure. So then, so they had to email me quickly. Give me, […] gave me half an hour to read them [report] and, […] to adjourn the meeting and give me half an hour to read them. Because the psychiatrist said she was going on holiday the next week. (P02, 405–408)

… all she [psychiatrist] said is he’s gonna be sectioned on a Section 3. I didn’t know the difference between Section 2, Section 3. I don’t know Mental Health Act. I don’t know social care. (P03, 108–110)

Admissions lasted several months and carers described their frustration at how slow it was for things to be actioned. In contrast, carers often experienced the discharge as something that came about suddenly. When sharing their stories, carers often tried to give times and dates to the various difficult events without being asked to do so, appearing to try and form a coherent narrative from a traumatic experience.

“*They might be mental health but they are not necessarily experts in dementia*”

A mental health admission was described by the professionals undertaking the section process to carers as a “specialist” placement. However, apparent during the interviews was that wards and units varied in their dementia specific focus. Given the variation in types of wards people were admitted to, the level of dementia knowledge varied considerably. One gentleman with a diagnosis of young onset dementia was admitted to generic “working age” mental health wards, his carer felt that staff lacked expertise and an of understanding of dementia;

He […] he loved plastic bags. So he took a plastic bag off the cleaning trolley and they restrained him to get it back, […] back and gave him Lorazepam to calm him down. So it’s like, […] I mean, […] I said to them, surely you understand dementia? You swap out something, you know, again, these are experts, […] supposedly. That’s what everyone keeps telling me in that setting, but they’re not. The might be mental health but they’re not necessarily in dementia, are they? And then they don’t seem to comprehend the differences in the different types of dementia either. You know, it’s individual to the person isn’t it? What might work for him, might not work for someone else. (P03, 462–470)

Staff shortages were mentioned in several interviews and this was seen to be one for poor communication with families. Families spoke about the high levels of distress that staff were trying to manage;

Every time I’d ring up, I’d ring up every day and there’d be this person saying, oh yeah, he’s been throwing chairs about how he’s scared of him. I had nurses crying on the phone to me. It’s just like I really did struggle with the fact that these meant, supposedly the experts from there, everybody kept saying to me he’s better in these systems. Any yeah they obviously couldn’t deal with him at all, you know. (P03, 346–351)

Two participants (a carer and his spouse) spoke positively about the staff working on the ward;

I have to say I thought the staff were wonderful, absolutely no complaints about them at all. (P05, 462–463)

We [carers spouse] felt that we could leave mum, we felt that they could take responsibility and were doing it well. (P04, 505–506)

“*There was just this lack of care*”

Three participants shared concerns about poor care, namely a failure to meet the person with dementia’s basic needs, this included not taking people to the toilet, not washing or showering people, not cutting hair or finger nails. Poor care was attributed to staff struggling to manage the person’s distress, a lack of care or concern about the person and restrictive practices on the ward, such as locked bathrooms and rigid routines;

They didn’t take [him] to toilet but he couldn’t so he peed on the floor. He didn’t have an en-suite bathroom. His door was locked, so he couldn’t access anything. They took his hearing aids away. They took his iPad’s away … had his tunes on and an alarm on it. (P03, 157–160)

These three carers spoke about the culture of care on the wards and of a lack of person centered care that was seen to arise from rigid ward routines, a lack of time taken to get to know the person and to explain things to them, all known elements of good dementia care;

… it’s individual to the person isn’t it? What might work for him, might not work for someone else. It is about approach, and it’s about tone of voice you know? Don’t go on too quickly. Don’t quick clap your hands around … it’s all those kind of things that you’ve got to put in place for it which just don’t seem to kind of be there really. (P03, 469–473)

… so they’d obviously mixed his clothes up with another patient right from day one, and it does so upset you. (P02, 810–814)

## 4. Discussion

This is the first study to explore family carers experiences of a mental health admission for their relative with dementia. The results highlight the support needs of family members during these lengthy admissions. Family carers were exhausted and in crisis and for the majority of carers, the admission added to their distress. Carers interviews were often jumbled and full of images and emotions, the interviewers were reminded of the narratives of those who had experienced trauma (20) and who struggle to assimilate and reconcile memories of what happened with their assumptions about the world and themselves (21). Findings suggest that carers could benefit from psychotherapeutic interventions while their relative with dementia is being cared for within a mental health ward. Psychosocial interventions to support family carers with depression and anxiety have a growing evidence base (22) and many wards will have clinical psychologists working within them who could be well placed to offer this support. Alternatively, a case management approach could be beneficial, where families have a source of contact who could ensure that problems are anticipated in order to offset crises (23).

Family members struggled with feelings of grief and guilt, and for some the subsequent sense of being excluded from the relatives care felt like a punishment for failing to care effectively and keep their relative at home. Wards varied significantly in the extent to which they involved families in their relatives care. It is not clear why some wards did not engage family members, although staffing shortages, pressures of time and risk management, rigid routines and attention to basic physical needs have been cited as dominating care on mental health wards (24). These environments have been characterized as distressing and unpredictable and the distressed behaviors and emotions present physical and emotional challenges to ward staff (24). Engaging families to learn more about the person with dementia, particularly when they are isolated from their usual environment, is essential to making sense of distress and behaviors that might seem unusual and essential to delivering good care (25).

Staff working in mental health with people with dementia have reported that relationships with families are often strained including tension and conflict with the potential for misunderstanding and negative interactions (26). This study suggests that some of this tension is likely to result from carer feelings of distress and guilt. As such, this research supports previous conclusions that the interface between families and the ward is an aspect that deserved greater input from management and from research (24).

Carers were concerned about the impact of the admission on their relative with dementia. Little is known about outcomes following mental health admission for people with dementia (8), future research should seek to use creative methods to engage people with dementia in sharing their views and experiences. Qualitative research conducted with care home residents with advanced dementia may provide some useful insights into inclusive methodologies (27).

Finally, it is important to acknowledge the concerns raised by three carers in relation to a lack of care and a lack of understanding about dementia on these wards. These concerns echoed the findings of historical investigations conducted in these settings (28, 29) and highlight the importance of conducting further research given the vulnerability of this population with dementia. As part of this study all carers raising concerns of poor practice were supported to escalate their concerns.

It is important to acknowledge the limitations of this small exploratory qualitative study. The aim of qualitative research is not to provide generalizable findings and as such the aim is not to tell the story of all carers of people with dementia admitted to mental health wards. Our aim is to promote more research and investigation. The volunteer nature of the sample could mean that those who wanted to take part were motivated to do so following a negative experience, indeed three participants had concerns about the quality of the care their relative had received. While being in hospital is challenging for people with dementia, an inpatient admission provides an opportunity for a holistic assessment. All carers had reached crisis prior to their relatives admission and recognized that urgent intervention was needed. Further research is needed with a larger sample to reflect more wards from across the United Kingdom and also to reflect the range of different types of mental health ward that people with dementia might be admitted to (e.g., experiences of specialist dementia wards vs. mixed old age wards). The sample of people with dementia these carers supported were largely people with young onset dementia and some had rare forms of dementia, which may not be typical of the general population of people with dementia admitted to mental health wards (30). Further research is needed to engage with carers of older people with dementia and the expansion of recruitment methods beyond online methods may support this. For four carers, their experience of admission was during the COVID pandemic which added to restrictions of visiting and staff pressures and is likely to have affected their perception of the admission.

## 5. Conclusion

The experiences of carers during their relative’s admission to a mental health ward indicate that they may benefit from therapeutic support. Wards need to recognize the expertise of carers and involve them in their assessment and interventions. Wards need to ensure that they have processes to communicate with families and should not assume any prior understanding of mental health law or terminology. More research is needed to explore the outcomes and experiences of a mental health admission for people with dementia.

## Data availability statement

The original contributions presented in the study are included in the article/supplementary materials, further inquiries can be directed to the corresponding author/s.

## Ethics statement

The studies involving human participants were reviewed and approved by University of Hull Research Ethics Committee in the Faculty of Health Sciences. The patients/participants provided their written informed consent to participate in this study.

## Author contributions

EW: designed the study, obtained ethical approval, collected data, analyzed data and drafted the manuscript. KHD: co-designed the study, collected data, analyzed data and supported writing the manuscript. RD: supported data collection, data analysis and the supported in writing the manuscript. GC, GR, and BU supported in designing the study and provided feedback and edits on the manuscript. All authors contributed to the article and approved the submitted version.

## Conflict of interest

The authors declare that the research was conducted in the absence of any commercial or financial relationships that could be construed as a potential conflict of interest.

The reviewer KDV declared a past collaboration with one of the authors KHD to the handling editor.

The reviewer AP declared a past collaboration with the authors KHD and EW to the handling editor.

## Publisher’s note

All claims expressed in this article are solely those of the authors and do not necessarily represent those of their affiliated organizations, or those of the publisher, the editors and the reviewers. Any product that may be evaluated in this article, or claim that may be made by its manufacturer, is not guaranteed or endorsed by the publisher.

## Appendix A: Interview guide

Interviews were semi-structured and explored the themes below:

- Expectations of inpatient care
- Information about an admission
- Communication with the ward
- Relationships with ward staff
- Involvement in care/treatment plans
- Perceptions of family support
- Experiences and involvement in discharge planning
- Satisfaction with mental health care
- Perceptions of outcomes (positive and negative) of the admission.
